# Mixture of experts models to exploit global sequence similarity on biomolecular sequence labeling

**DOI:** 10.1186/1471-2105-10-S4-S4

**Published:** 2009-04-29

**Authors:** Cornelia Caragea, Jivko Sinapov, Drena Dobbs, Vasant Honavar

**Affiliations:** 1 Artificial Intelligence Research Laboratory, Computer Science Department, Iowa State University, Ames, IA, 50010, USA; 2 Center for Computational Intelligence, Learning, and Discovery, Iowa State University, Ames, IA, 50010, USA; 3 Department of Genetics, Development and Cell Biology, Iowa State University, Ames, IA, 50010, USA; 4 Bioinformatics and Computational Biology Program, Iowa State University, Ames, IA, 50010, USA

## Abstract

**Background:**

Identification of functionally important sites in biomolecular sequences has broad applications ranging from rational drug design to the analysis of metabolic and signal transduction networks. Experimental determination of such sites lags far behind the number of known biomolecular sequences. Hence, there is a need to develop reliable computational methods for identifying functionally important sites from biomolecular sequences.

**Results:**

We present a *mixture of experts *approach to biomolecular sequence labeling that takes into account the global similarity between biomolecular sequences. Our approach combines unsupervised and supervised learning techniques. Given a set of sequences and a similarity measure defined on pairs of sequences, we learn a mixture of experts model by using spectral clustering to learn the hierarchical structure of the model and by using bayesian techniques to combine the predictions of the experts. We evaluate our approach on two biomolecular sequence labeling problems: RNA-protein and DNA-protein interface prediction problems. The results of our experiments show that global sequence similarity can be exploited to improve the performance of classifiers trained to label biomolecular sequence data.

**Conclusion:**

The *mixture of experts *model helps improve the performance of machine learning methods for identifying functionally important sites in biomolecular sequences.

## Background

Advances in high throughput data acquisition technologies have resulted in rapid increase in the amount of data in biological sciences. For example, progress on sequencing technologies has resulted in the release of hundreds of complete genome sequences. With the exponentially growing number of biomolecular sequences from genome projects and high-throughput experimental studies, sequence annotations do not keep pace with sequencing.

The wet-lab experiments to determine the annotations (e.g., functional site annotations) are still difficult and time consuming. Hence, there is an urgent need for development of computational tools that can accurately annotate biomolecular data.

Machine learning methods currently offer one of the most cost-effective approaches to construction of predictive models in applications where representative training data are available. *Biomolecular sequence labeling *is an instance of a supervised learning problem. Given a data set (**x**_*i*_, **y**_*i*_)_*i *= 1,⋯, *n *_of pairs of sequences, **x**_*i *_= (*x*_*i*,1 _*x*_*i*,2 _⋯*x*_*i, m*_) and **y**_*i *_= (*y*_*i*,1 _*y*_*i*,2 _⋯*y*_*i, m*_), where *y*_*i, j *_in the output sequence is the label for *x*_*i, j *_in the input (or observation) sequence, *j *= 1,⋯, *m*, the task is to learn a classifier that can predict the labels for each element of a new input sequence, **x**_*test*_.

There is a large body of work on learning predictive models to label biomolecular sequence data. Terribilini *et al*. [1] trained Naïve Bayes classifiers to identify RNA-protein interface residues in a protein sequence. Yan *et al. *[2] developed a two-stage classifier to identify protein-protein interaction sites. Qian and Sejnowski [3] trained Neural Networks to predict protein secondary structure, i.e., classifying each residue in a protein sequence into one of the three classes: helix (H), strand (E) or coil (C). Caragea *et al. *[4] and Kim *et al. *[5] used Support Vector Machines to identify residues in a protein sequence that undergo post-translational modifications.

Typically, to solve the biomolecular sequence labeling problem using standard machine learning algorithms, each element in a sequence is encoded using a local, fixed-length window corresponding to the target element and its sequence context (an equal number of its sequence neighbors on each side) [6]. The classifier is trained to label the target element. This procedure can produce reliable results in settings where there exists a local sequence pattern that is predictive of the label for the target site. However, there are cases where the local amino acid distribution around functionally important sites in a given set of proteins is highly variable. For example, in identifying RNA-protein and DNA-protein interface residues from amino acid sequences, there is typically no consensus sequence around each site.

Classifiers trained using machine learning to distinguish "positive" examples from the "negative" ones, must "learn" to do so by learning the characteristics associated with known "positive" and "negative" examples. The greater the commonality among members of a subset, the more likely it is that a machine learning approach will be successful in identifying the predictive characteristics.

Against this background, we hypothesize that classifiers trained to label biomolecular sequence data can be improved by taking into account the global sequence similarity between the protein sequences in addition to the local features extracted around each site. The intuition behind this hypothesis is that the more similar two sequences are, the greater the likelihood that their functional sites have similar patterns. Therefore, we propose to improve the biomolecular sequence labeling problem by using a machine learning approach, that is, a *mixture of experts *model that considers the global similarity between protein sequences when building the model and making the predictions. We evaluate our approach to learning a *mixture of experts *model on two biomolecular sequence labeling tasks: RNA- and DNA-protein interface prediction tasks.

## Results

The main result of our study is that taking into account global sequence similarity through the means of a *mixture of experts *model can improve the performance of the classifiers trained to label biomolecular sequence data.

### The mixture of experts that exploits the global similarity between the protein sequences in a data set in addition to the local features extracted around each residue outperforms the baseline classifiers on the biomolecular sequence labeling task

We trained mixtures of Naïve Bayes (NB) and Logistic Regression (LR) classifiers on both RNA- and DNA-protein interface prediction tasks considered in this study to predict whether or not a residue in a protein sequence is an interface residue. We used various identity cutoffs to construct the data sets. The mixture of experts models have a hierarchical structure that is constructed using 2-way spectral clustering based on a global similarity functions, i.e., we computed the entries in the similarity matrix **W **by applying the Needleman-Wunsch global alignment algorithm on each pair of sequences. The Blosum62 substitution matrix was used for costs. The resulting entries in the matrix **W **are normalized and scaled so that each value is between 0 and 1. The mixture of experts models consist of NB and LR at the leaves, respectively (see Methods section for further details).

**Figure 1:**
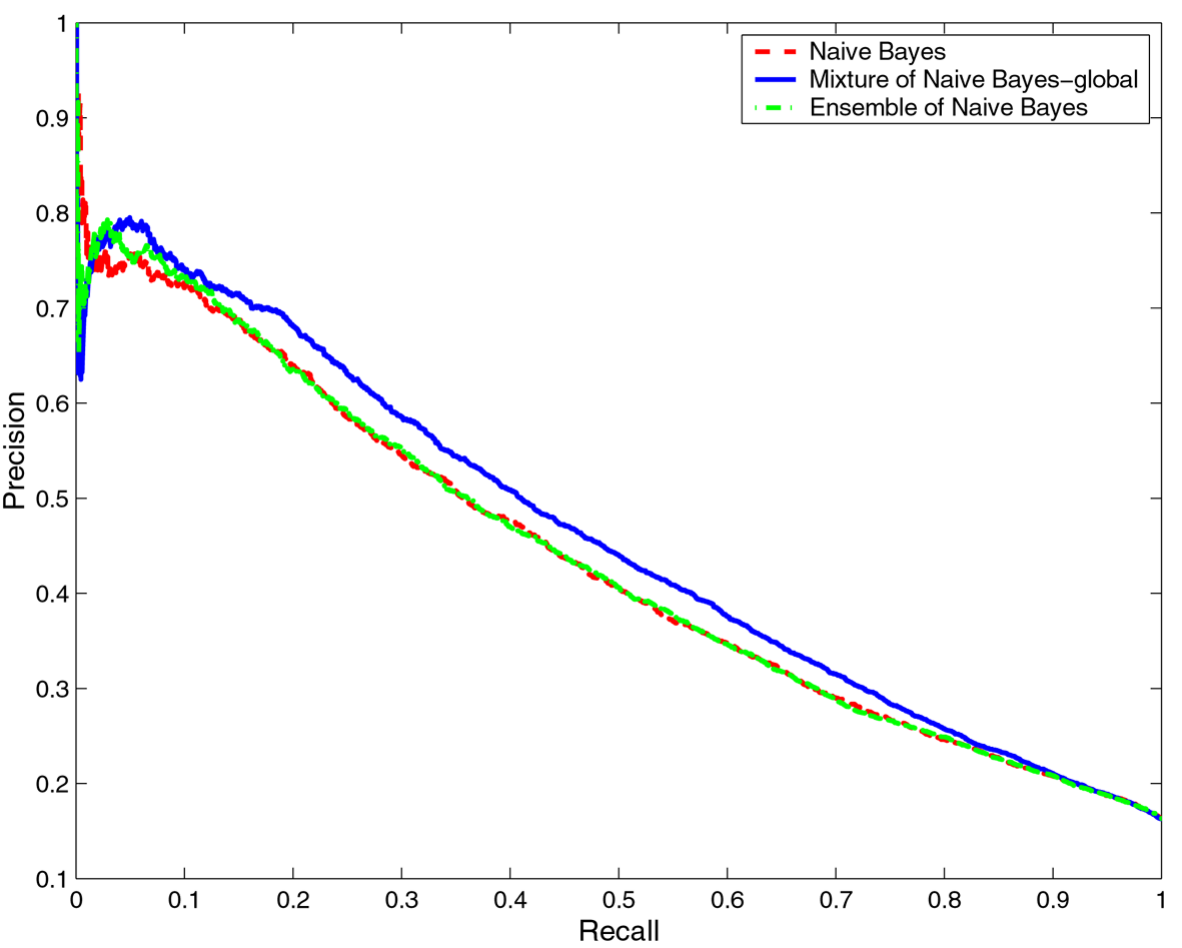
**Comparison of Naïve Bayes, mixture of Naïve Bayes and ensemble of Naïve Bayes models on the RNA-protein data set**. Comparison of Precision-Recall curves for Naïve Bayes, mixture of Naïve Bayes and ensemble of Naïve Bayes models on the non-redundant RNA-protein data set at 30% identity cutoff. The hierarchical structure of the mixture of experts model is constructed based on global sequence similarity.

**Figure 2:**
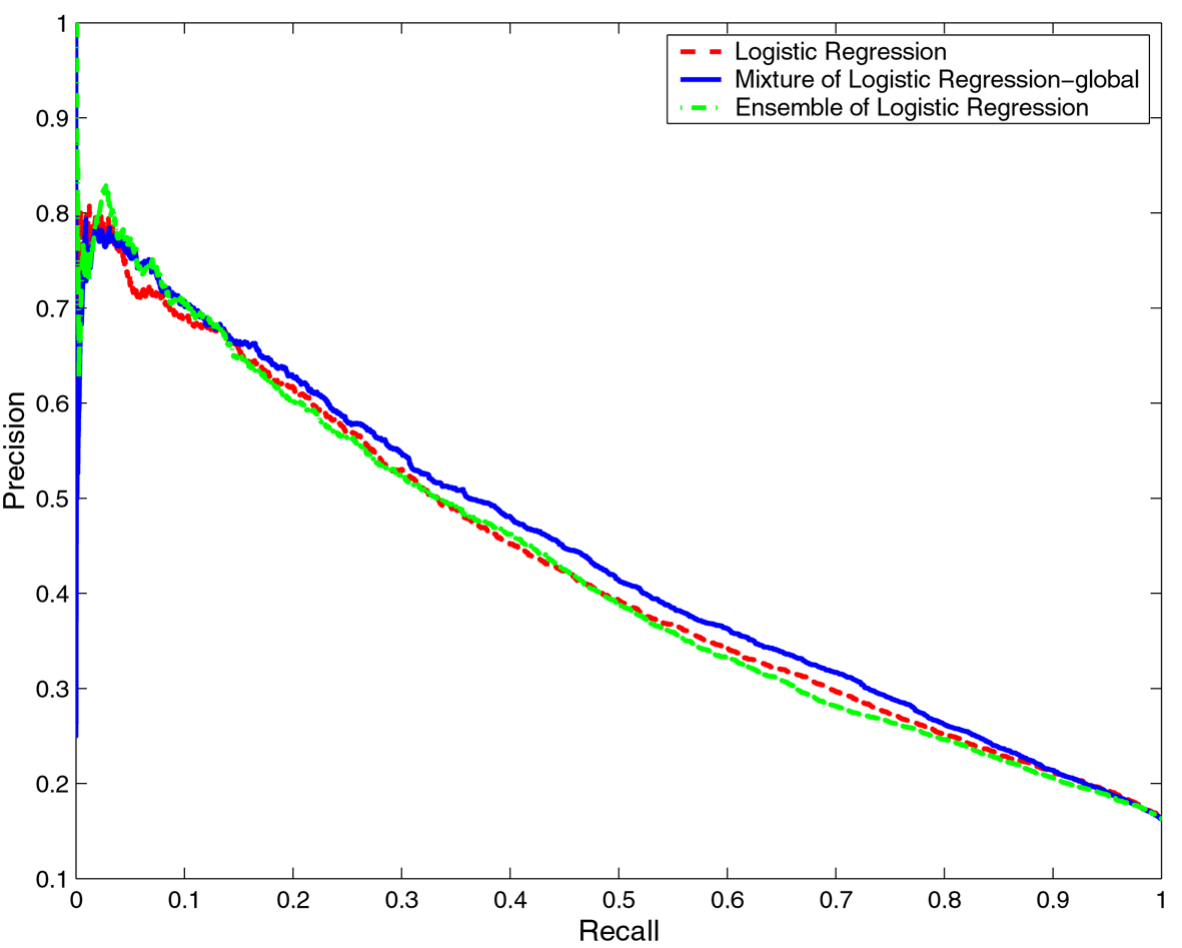
**Comparison of Logistic Regression, mixture of Logistic Regression and ensemble of Logistic Regression models on the RNA-protein data set**. Comparison of Precision-Recall curves for Logistic Regression, mixture of Logistic Regression and ensemble of Logistic Regression models on the non-redundant RNA-protein data set at 30% identity cutoff. The hierarchical structure of the mixture of experts model is constructed based on global sequence similarity.

**Figure 3:**
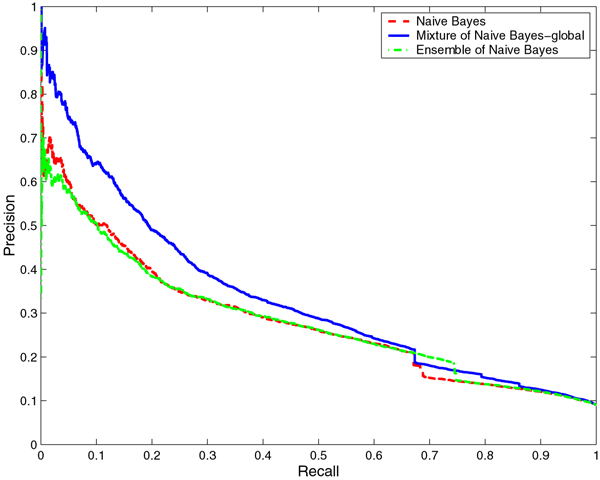
**Comparison of Naïve Bayes, mixture of Naïve Bayes and ensemble of Naïve Bayes models on the DNA-protein data set**. Comparison of Precision-Recall curves for Naïve Bayes, mixture of Naïve Bayes and ensemble of Naïve Bayes models on the non-redundant DNA-protein data set at 30% identity cutoff. The hierarchical structure of the mixture of experts model is constructed based on global sequence similarity.

**Figure 4:**
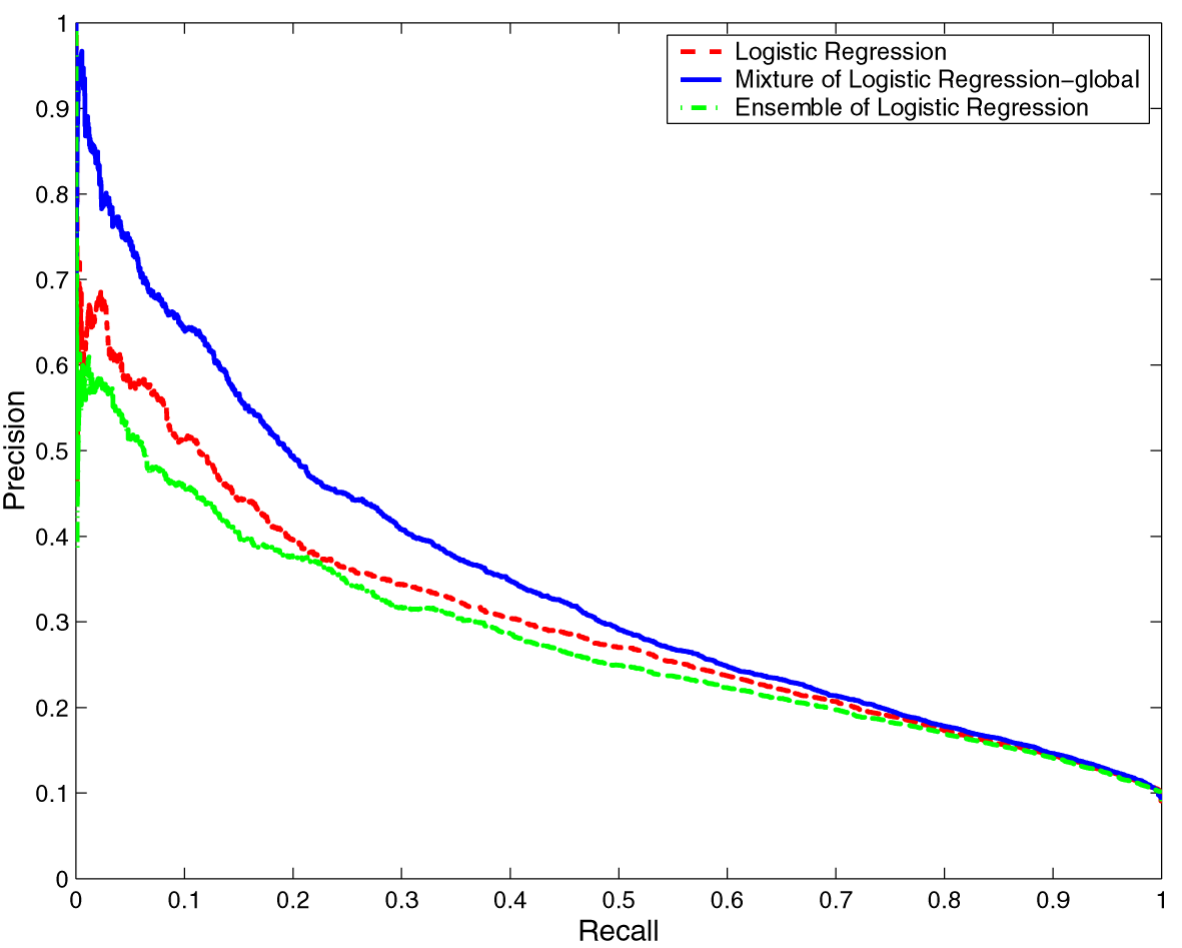
**Comparison of Logistic Regression, mixture of Logistic Regression and ensemble of Logistic Regression models on the DNA-protein data set**. Comparison of Precision-Recall curves for Logistic Regression, mixture of Logistic Regression and ensemble of Logistic Regression models on the non-redundant DNA-protein data set at 30% identity cutoff. The hierarchical structure of the mixture of experts model is constructed based on global sequence similarity.

We compared the performance of the mixtures of NB and LR with that of baseline NB and LR, respectively. With any classifier, it is possible to tradeoff the Precision against Recall. Hence, it is more informative to compare the Precision-Recall (PR) curves which show the tradeoff over their entire range of possible values than to compare the performance of the classifiers for a particular choice of the tradeoff. Thus, we compared the PR curves for NB and the mixture of NB models as well as LR and mixture of LR models on both RNA- and DNA-protein interface prediction tasks. For both prediction tasks, the PR curves for the mixture of experts models dominate the PR curves of NB and LR models, that is, for any choice of Recall, the mixture of experts models offer a higher Precision than NB and LR (Figures 1, 2, 3, and 4 respectively). While this is true for any identity cutoff for both RNA- and DNA-protein sequence data sets, we show results only for 30% identity cutoff. The curves demonstrate that even for a very stringent cutoff, the mixture of experts that captures global similarity between sequences in the data set outperforms the other models.

In Tables 1 and 2, we show the classification results after evaluating the baseline models, NB and LR, and the mixture of experts models with NB and LR at the leaves, ME-NB-global and ME-LR-global, respectively, on the RNA- and DNA-protein sequence data sets for two identity cutoffs: 30% and 90%. The values in the tables are obtained using the default threshold *θ *= 0.5. As illustrated in the tables, the mixture of experts models that capture global sequence similarity outperform the baseline models. For example, in the case of RNA-protein data set at 30% identity cutoff, the mixture of experts, ME-NB-global, achieves 0.61 Precision, 0.27 Recall, 0.34 Correlation Coefficient, 0.38 F-Measure, and 0.77 Area Under the ROC Curve, while the NB classifier achieves 0.58 Precision, 0.25 Recall, 0.31 Correlation Coefficient, 0.35 F-Measure, and 0.75 Area Under the ROC Curve (Table 1). In the case of DNA-protein data set at 30% identity cutoff, the mixture of experts, ME-NB-global, achieves 0.62 Precision, 0.12 Recall, 0.25 Correlation Coefficient, 0.20 F-Measure, and 0.77 Area Under the ROC Curve, while the NB classifier achieves 0.59 Precision, 0.05 Recall, 0.16 Correlation Coefficient, 0.10 F-Measure, and 0.75 Area Under the ROC Curve (Table 2).

**Table 1:** Experimental results on the RNA-protein sequence data set. Experimental results with Naive Bayes (NB) and Logistic Regression (LR) models, and Mixture of Experts (ME) models on the non-redundant RNA-protein sequence data set, where the identity cutoffs are 30% and 90%. The results are shown for default threshold *θ *= 0.5. ME-NB-global and ME-LR-global use NB and LR at the leaves and exploits the global sequence similarity to construct the hierarchical structure. ME-NB-local exploits the local sequence similarity to construct the hierarchical structure. ME-NB-random randomizes the global similarity matrix and constructs the hierarchical structure based on the randomized matrix.

	RNA-protein 30%					RNA-protein 90%				
Classifier	Precision	Recall	CC	FM	AUC	Precision	Recall	CC	FM	AUC

NB	0.58	0.25	0.31	0.35	0.75	0.58	0.30	0.33	0.40	0.77
ME-NB-global	0.61	**0.27**	**0.34**	**0.38**	**0.77**	**0.61**	**0.32**	**0.36**	**0.42**	**0.78**
ME-NB-local	**0.62**	0.25	0.33	0.35	0.76	**0.61**	0.30	0.34	0.40	0.77
ME-NB-random	0.59	0.24	0.31	0.35	0.75	0.59	0.30	0.33	0.40	0.77

LR	**0.62**	0.18	0.28	0.29	0.76	**0.63**	0.23	0.31	0.34	0.77
ME-LR-global	0.60	**0.23**	**0.31**	**0.34**	**0.77**	0.61	**0.27**	**0.33**	**0.38**	**0.78**

**Table 2:** Experimental results on the DNA-protein sequence data set. Experimental results with Naive Bayes (NB) and Logistic Regression (LR) models, and Mixture of Experts (ME) models on the non-redundant DNA-protein sequence data set, where the identity cutoffs are 30% and 90%. The results are shown for default threshold *θ *= 0.5. ME-NB-global and ME-LR-global use NB and LR at the leaves and exploits the global sequence similarity to construct the hierarchical structure. ME-NB-local exploits the local sequence similarity to construct the hierarchical structure. ME-NB-random randomizes the global similarity matrix and constructs the hierarchical structure based on the randomized matrix.

Classifier	DNA-protein 30%					DNA-protein 90%				
	Precision	Recall	CC	FM	AUC	Precision	Recall	CC	FM	AUC

NB	0.59	0.05	0.16	0.10	0.75	0.56	0.07	0.18	0.13	0.75
ME-NB-global	0.62	**0.12**	**0.25**	**0.20**	**0.77**	**0.65**	**0.15**	**0.29**	**0.25**	**0.78**
ME-NB-local	**0.65**	0.06	0.18	0.12	0.76	0.64	0.08	0.21	0.15	0.76
ME-NB-random	0.58	0.05	0.15	0.09	0.75	0.56	0.07	0.18	0.13	0.75

LR	0.57	0.07	0.18	0.12	0.79	0.57	0.08	0.18	0.14	0.79
ME-LR-global	**0.57**	**0.14**	**0.26**	**0.23**	**0.80**	**0.63**	**0.17**	**0.29**	**0.26**	**0.81**

### The mixture of experts that exploits the global similarity between protein sequences outperforms a mixture of experts that exploits the local similarity between protein sequences

In order to verify that indeed global sequence similarity is helpful in improving the performance of classifiers, and that the improvement does not come from the more sophisticated structure of the model, we computed the entries in the similarity matrix **W **by applying Smith-Waterman local alignment algorithm with Blosum62, thus taking into account local sequence similarity (the matrix **W **is normalized and scaled as before). We also randomized the global similarity matrix computed previously and use this randomized matrix to construct the hierarchical structure of the mixture of experts models.

In Tables 1 and 2 we show the performance of NB and mixture of NB models using global (ME-NB-global) and local (ME-NB-local) sequence similarities, as well as a random (ME-NB-random) sequence similarity for the default threshold *θ *= 0.5. The results of our experiments show that the mixture of experts models that capture global sequence similarity outperform the other models in terms of the majority of standard measures for comparing the performance of classifiers used in this study (the results are similar for the mixture of LR models, data not shown). For example, for 30% identity cutoff, Correlation Coefficient increases from 0.33 (local similarity) to 0.34 (global similarity) on the RNA-protein data set (Table 1), and from 0.18 (local similarity) to 0.25 (global similarity) on the DNA-protein data set (Table 2). Hence, we conclude that global similarity is helpful in improving the performance of classifiers trained to label biomolecular sequence data.

### The mixture of experts has consistently higher performance than the baseline classifier for all identity cutoffs

**Figure 5:**
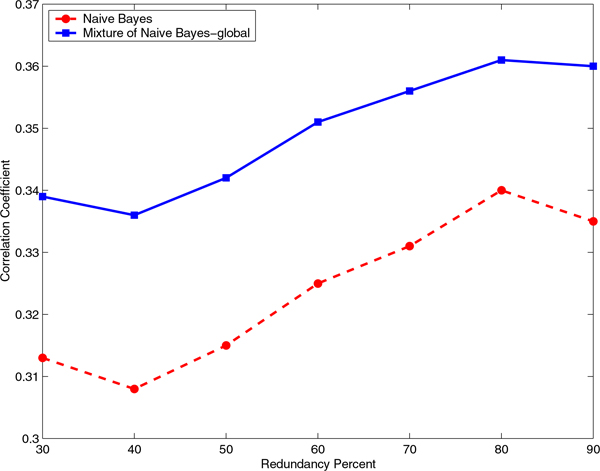
**Comparison of Correlation Coefficient for Naïve Bayes and mixture of Naïve Bayes models on the RNA-protein data set**. Comparison of Correlation Coefficient for Naïve Bayes and mixture of Naïve Bayes models that capture global sequence similarity on the non-redundant RNA-protein data sets constructed using various identity cutoffs, starting from 30% and ending at 90% in steps of 10.

**Figure 6:**
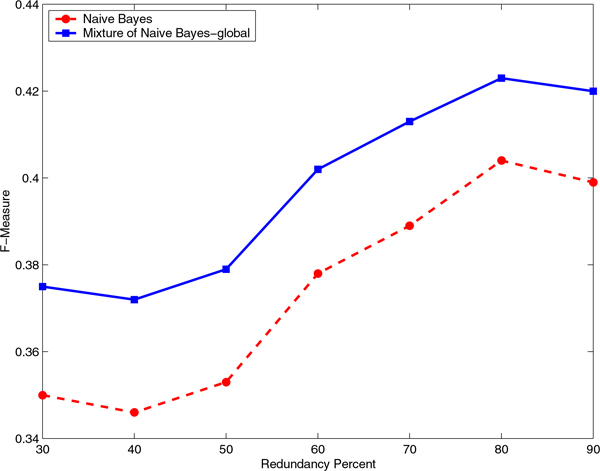
**Comparison of F-Measure for Naïve Bayes and mixture of Naïve Bayes models on the RNA-protein data set**. Comparison of F-Measure for Naïve Bayes and mixture of Naïve Bayes models that capture global sequence similarity on the non-redundant RNA-protein data sets constructed using various identity cutoffs, starting from 30% and ending at 90% in steps of 10.

**Figure 7:**
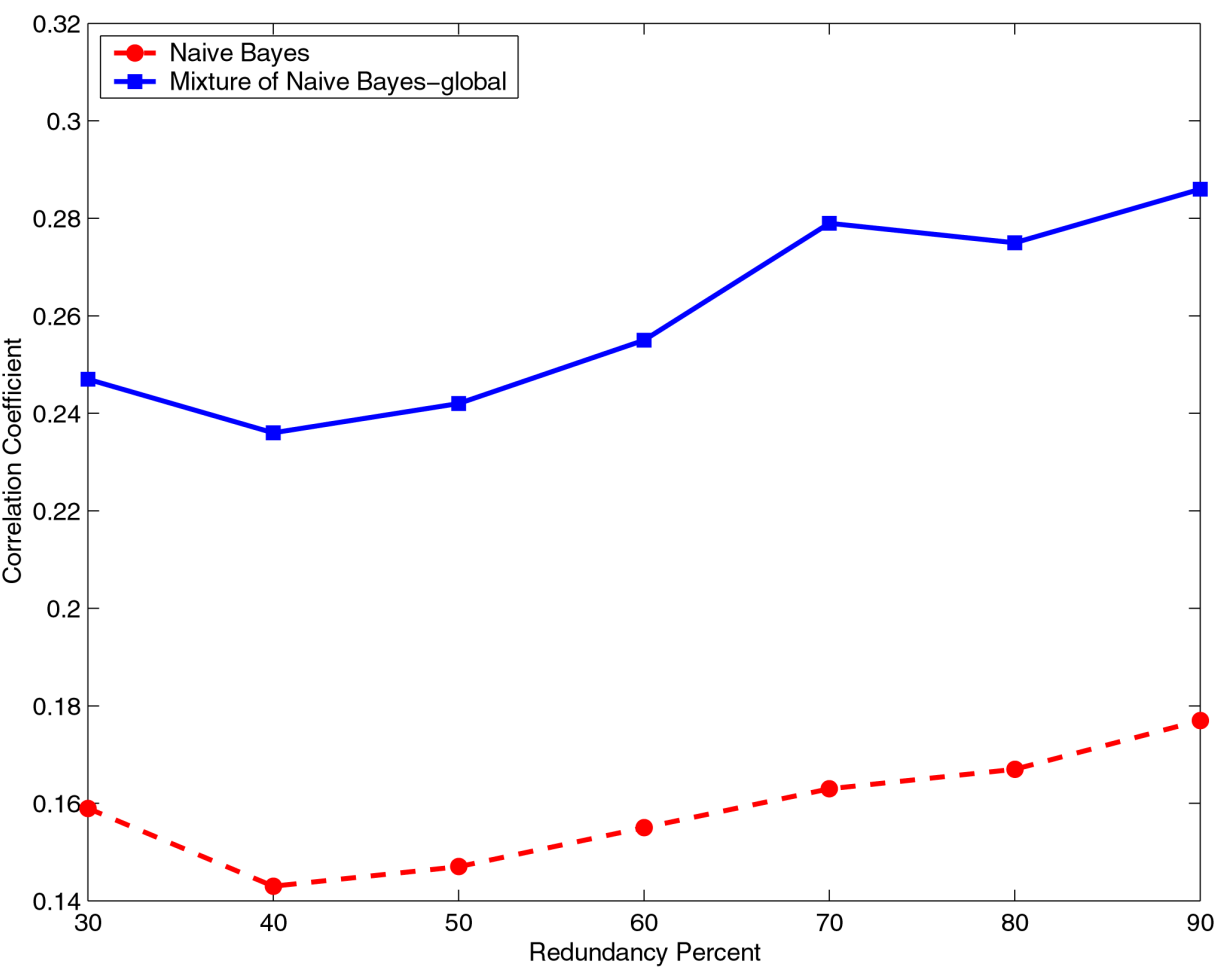
**Comparison of Correlation Coefficient for Naïve Bayes and mixture of Naïve Bayes models on the DNA-protein data set**. Comparison of Correlation Coefficient for Naïve Bayes and mixture of Naïve Bayes models that capture global sequence similarity on the non-redundant DNA-protein data sets constructed using various identity cutoffs, starting from 30% and ending at 90% in steps of 10.

**Figure 8:**
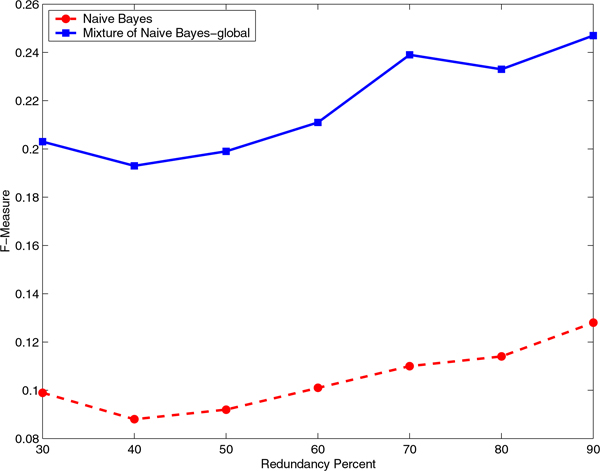
**Comparison of F-Measure for Naïve Bayes and mixture of Naïve Bayes models on the DNA-protein data set**. Comparison of F-Measure for Naïve Bayes and mixture of Naïve Bayes models that capture global sequence similarity on the non-redundant DNA-protein data sets constructed using various identity cutoffs, starting from 30% and ending at 90% in steps of 10.

We evaluated the effect of the identity cutoff to construct the non-redundant data sets on the Correlation Coefficient and F-Measure for a range of sequence identity cutoffs from 30% to 90% (Figures 5, 6, 7, and 8). It is interesting to note that even at a very stringent sequence identity cutoff of 30% the difference in the Correlation Coefficient and the difference in the F-Measure for the mixture of experts and the baseline classifiers is significant, on both RNA- and DNA-protein data sets.

### The mixture of experts that exploits the global sequence similarity offers a higher precision than the ensemble of classifiers for the same Recall

We also trained ensembles of NB and LR classifiers on both RNA- and DNA-protein interface prediction tasks to predict whether or not a residue in a protein sequence is an interface residue. An ensemble of classifiers [7,8] is simply a collection of classifiers, each trained on a *balanced *subsample of the training data. The prediction of the ensemble is computed from the predictions of the individual classifiers (see Methods section for further details).

We compared the performance of the mixtures of NB and LR with that of ensembles of NB and LR, respectively. In Figures 1, 2, 3, and 4 we show the PR curves for the mixture and the ensemble models on both RNA- and DNA-protein sequence data sets for 30% identity cutoff. As can be seen from the figures, the mixtures of experts consistently offer a higher Precision than the ensembles of classifiers for the same Recall. Note that the PR curves of the ensembles are closer to those of the baseline classifiers.

## Discussion

Reliable methods for identifying putative functional sites in protein sequences is an important problem with broad applications in computational biology, e.g., rational drug design. Computational tools for identifying functional sites from *sequences *are especially important because of the cost and efforts involved in structure determination.

In this work we sought to improve the performance of classifiers that make predictions on residues in protein sequences by taking into account the global similarity between the protein sequences in the data set in addition to the local features extracted around each residue. We evaluated *mixture of experts *models that consider the global similarity between protein sequences when building the model and making the predictions on the RNA-protein and DNA-protein interface prediction tasks. Two closely related models are the Hierarchical Mixture of Experts model [9] and the ensemble of classifiers model [7]

### Hierarchical Mixture of Experts

The Hierarchical Mixture of Experts model (HME) was first proposed by Jordan and Jacobs (1994) [9] to solve nonlinear classification and regression problems by combining linear models: the input space is divided into a set of nested regions and simple (e.g., linear) models are fit to the data that fall in these regions. Hence, instead of using a "hard" partitioning of the data, the authors use a "soft" partitioning, i.e., the data is allowed to simultaneously lie in more than one region.

The HME has a tree-structured architecture that is known *a priori*. The internal nodes of the tree correspond to *gating networks *and the leaf nodes correspond to *expert networks*. The expert networks output class probabilities for each input *x*, while the gating networks learn how to combine the predictions of the experts up the tree with the final prediction output by the root. The parameters of the gating networks are learned using Expectation Maximization algorithm [10]. The gating and the expert networks are generalized linear models.

### Ensemble of classifiers

An ensemble of classifiers is a collection of independent classifiers, each classifier being trained on a subsample of the training data [7]. The prediction of the ensemble of classifiers is computed from the predictions of the individual classifiers using majority voting. An example is misclassified by the ensemble if a majority of the classifiers misclassifies it. When the errors made by the individual classifiers are uncorrelated, the predictions of the ensemble of classifiers are often more reliable.

### Mixture of experts – our approach

Our approach to learning a *mixture of experts *model takes into account the global similarity between biomolecular sequences in a data set. Unlike the HME model [9], we assume that the structure of our model is not known *a priori*. Hence, to learn the hierarchical structure of the model, we use hierarchical clustering of the sequences in the data set. The leaf nodes consist of expert classifiers, while the gating nodes combine the output of each classifier to the root of the tree which makes the final prediction. The gating nodes combine the predictions of the expert classifiers based on an estimate of the cluster membership of a test protein sequence. Following the approach taken by Jordan and Jacobs [9], we considered a "soft" partitioning of the data, i.e., each sequence in the training set simultaneously lies in all clusters of the hierarchical structure with a different weight in each cluster. The combination scheme of the predictions of the expert classifiers and the "soft" partitioning of the data that considers the global sequence similarity differentiate our model from an ensemble of classifiers model.

## Conclusion

Identification of functionally important sites in biomolecular sequences has broad applications ranging from rational drug design to the analysis of metabolic and signal transduction networks. With the rapid increase in the amount of data (e.g., protein sequences) there is a growing need for reliable procedures to accurately identify such sites.

In this study, we have presented a *mixture of experts *approach to identification of functionally important sites from amino acid sequence of proteins that takes into account global similarity between the protein sequences. Specifically, we systematically evaluated Naive Bayes and Logistic Regression classifiers, as well as mixtures of Naive Bayes and Logistic Regression in a sequence-based 10-fold cross-validation setup. The results of our experiments show that global sequence similarity through the means of the mixture of experts approach can be exploited to improve the performance of classifiers trained to label biomolecular sequence data.

## Methods

### Data sets and parameter settings

We used two datasets to perform experiments: **RNA-protein **and **DNA-protein interface **data sets that are available online at . RNA- and DNA-protein interactions play a pivotal role in protein function. Reliable identification of such interaction sites from protein sequences has broad applications ranging from rational drug design to the analysis of metabolic and signal transduction networks.

The RNA- and DNA-protein interface data sets consist of RNA- and DNA-binding protein sequences, respectively, extracted from structures in the Protein Data Bank (PDB) [11]. We downloaded all the protein structures of known RNA- and DNA-protein complexes from PDB solved by X-ray crystallography and having X-ray resolution between 0 and 3.5 Å. As of May 2008, the number of RNA-protein complexes was 435 and DNA-protein complexes was 1259. A residue was identified as interface residue using Entangle with the default parameters [12].

Furthermore, to remove redundancy in each data set, we used BlastClust, a toolkit that clusters sequences with statistically significant matches, available at [13]. While constructing our non-redundant sequence data sets, we applied various identity cutoffs, starting from 30% and ending at 90% in steps of 10. For example, in the 30% identity cutoff sequence data set, two sequences were pairwise matched if they were 30% or more identical over an area covering 90% of the length of each sequence. We randomly selected a sequence from each cluster returned by BlastClust. Thus, the resulting non-redundant RNA-protein sequence data set for 30% identity cutoff has 180 protein sequences. The total number of amino acid residues is 33,235.

We represented residues identified as interface residues in a protein sequence as positive instances (+) and those not identified as interface residues as negative instances (-). Furthermore, we encoded each residue by a local window of fixed length, winLength = 21, corresponding to the target residue and ten neighboring residues on each side.

Table 3 shows the number of sequences as well as the number of positive (+) and negative (-) instances in the non-redundant RNA- and DNA-protein sequence data sets for 30%, 60%, and 90% identity cutoffs. It is interesting to note that many sequences in both RNA- and DNA-protein interface data sets share 90% or greater sequence identity with one or more sequences in the respective data sets. When such sequences are removed from the data sets, the number of sequences reduces from 435 to 246 in the case of RNA-protein interface data set, and from 1259 to 317 in the case of DNA-protein interface data set. More stringent sequence identity cutoffs (e.g., 30%) do not result in a significant reduction in the size of the data sets.

**Table 3:** Number of sequences, as well as positive and negative instances used in our experiments for the RNA- and DNA-protein data sets. Number of sequences as well as number of positive (+) and negative (-) instances in the non-redundant RNA- and DNA-protein sequence data sets for 30%, 60%, and 90% identity cutoffs.

Data Sets	Number of Sequences	Number of + Instances	Number of - Instances
RNA-prot 30%	180	5398	27837

RNA-prot 60%	215	6689	32073

RNA-prot 90%	246	7798	34675

DNA-prot 30%	257	5326	53494

DNA-prot 60%	289	5974	58031

DNA-prot 90%	317	6551	60877

### Learning mixture of experts models

Here we present our approach to learning a *mixture of experts *model that takes into account the global similarity between biomolecular sequences. Unlike the Hierarchical Mixture of Experts model [9], we assume that the structure of our model is not known *a priori*. Hence, to learn the hierarchical structure of the model, we use hierarchical clustering of the sequences in the data set. The leaf nodes consist of expert classifiers, while the gating nodes combine the output of each classifier to the root of the tree which makes the final prediction. The gating nodes combine the predictions of the expert classifiers based on an estimate of the cluster membership of a test protein sequence. Similar to Jordan and Jacobs [9], we considered a "soft" partitioning of the data, i.e., each sequence in the training set simultaneously lies in all clusters of the hierarchical structure with a different weight in each cluster.

#### Learning the structure of the mixture of experts model

**Figure 9:**
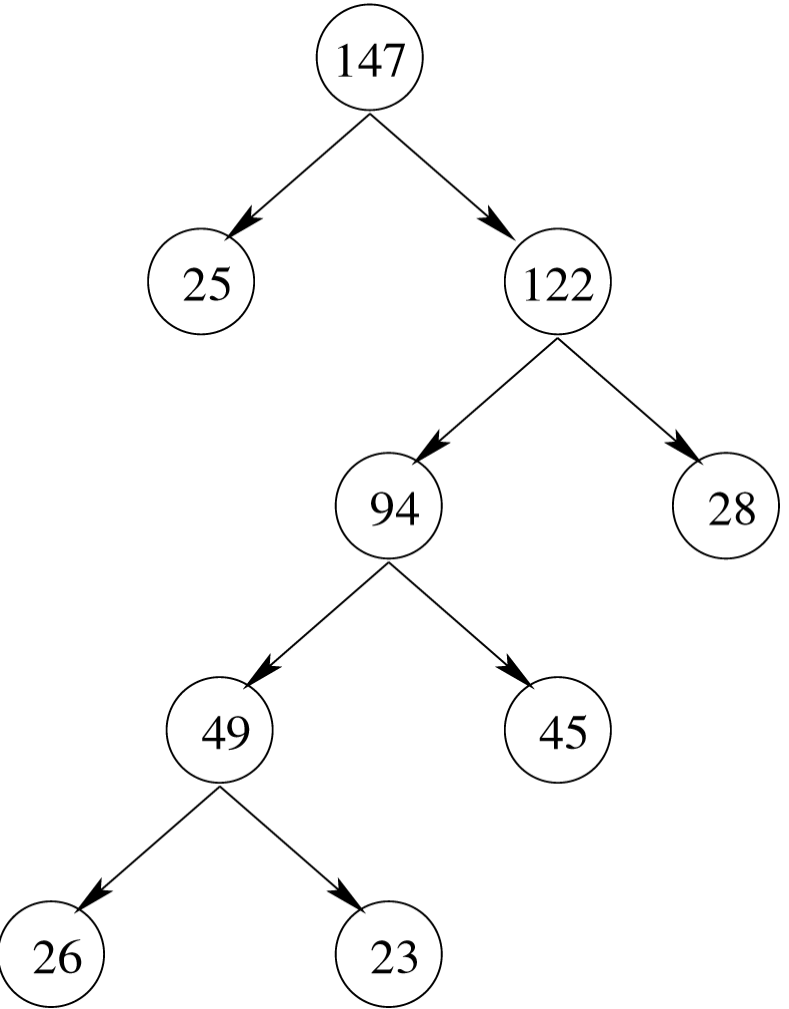
**Hierarchical structure produced by spectral clustering on a data set of 147 protein sequences**. The resulting hierarchical structure produced by spectral clustering when applied to a set of 147 RNA-protein sequences. The number in each node indicates the number of protein sequences belonging to it. The Needleman-Wunch global alignment score was used as a pairwise similarity measure during the clustering process.

To learn the hierarchical structure of our model, we use hierarchical clustering, an unsupervised learning technique [14] that attempts to uncover the hidden structure that exists in the unlabeled data. Given a data set D of unlabeled protein sequences (**x**_*i*_)_*i *= 1,..., *n*_, and a similarity measure *S *defined on pairs of sequences, the clustering algorithm *C *partitions the data into dissimilar clusters of similar sequences producing a tree-structured architecture (see Figure 9).

We first compute the pairwise similarity matrix **W**_*n *× *n *_for the protein sequences in the training set based on a common global sequence alignment method. Second, using this similarity matrix, we apply 2-way spectral clustering algorithm, described in the next subsection, to recursively bipartition the training set of protein sequences until a splitting criterion is met.

The output of the algorithm is a hierarchical clustering of the protein sequences, i.e., a tree T such that each node (cluster) consists of a subset of sequences. The root node is the largest cluster containing all the protein sequences in the training set. Once a cluster is partitioned into its two subclusters, it becomes their parent in the resulting tree structure. We store all the intermediate clusters computed by the algorithm. If the number of sequences at a given cluster falls below some percentage of the total sequences in the training set, then the node becomes a leaf and thus is not further partitioned (we used 10% in our experiments).

Figure 9 shows the tree structure produced by the 2-way spectral clustering algorithm when applied to a set of 147 RNA-protein sequences. The similarity matrix is computed based on the Needleman-Wunsch global alignment algorithm. In the figure, to keep the tree smaller, we stopped bipartitioning a node when the number of sequences at a given cluster falls below 30% of the total sequences in the training set.

#### 2-Way spectral clustering

Spectral clustering has been successfully applied in many applications, including image segmentation [15], document clustering [16], grouping related proteins according to their structural SCOP classification [17].

Spectral clustering falls within the category of graph partitioning algorithms that partition the data into disjoint clusters by exploiting the eigenstructure of a similarity matrix. In general, to find an optimal graph partitioning is NP complete. Shi and Malik [15] proposed an approximate spectral clustering algorithm that optimizes the *normalized cut *(NCut) objective function. It is a divisive, hierarchical clustering algorithm that recursively bi-partitions the graph until some criterion is reached, producing a tree structure.

Let X = {**x**_1_, **x**_2_,⋯, **x**_*n*_} be the set of sequences to be partitioned and let *S *be a similarity function between pairs of sequences. The 2-way spectral clustering algorithm consists of the following steps:

1. Let **W**_*n *× *n *_= [*S*(*i, j*)] be the symmetrical matrix containing the similarity score for each pair of sequences.

2. Let **D**_*n *× *n *_be the degree matrix of **W**, i.e., a diagonal matrix such that **D**_*ii *_= ∑_*j *_*S*(*i, j*).

3. Solve the eigenvalue system (**D **- **W**)*x *= *λ ***D***x *for the eigenvector corresponding to the second smallest eigenvalue and use it to bipartition the graph.

4. Recursively bipartition each subgraph obtained at Step 3. if necessary.

Note that the quality of the clusters found by the 2-way spectral clustering algorithm depends on the choice of the similarity function *S*.

#### Estimating the parameters of the mixture of experts model

Following the approach taken by Jordan and Jacobs [9], we make use of the "soft" partitioning of the biomolecular sequence data. Thus, having the hierarchical clustering T stored, we devise a procedure that allows each sequence in the training set to simultaneously lie in all clusters, with a different weigth in each cluster.

For each sequence **x**_*i*_, *i *= 1,⋯, *n *in the training set D, we compute its cluster membership as follows:

1. Find the *K *closest sequences to **x**_*i *_at the parent node based on the similarity function used to construct the hierarchical clustering T (in our experiments we used *K *equal to 20% of the sequences at the parent node).

2. Let *K*_0 _out of *K *sequences go to the left child node, and *K*_1 _out of *K *go to the right child node.

3. The estimated probability of **x**_*i *_for being in child node *j *is computed as *p*(**x**_*i *_∈ *V*_*j*_|**x**_*i *_∈ *par*(*V*_*j*_)) = *K*_*j*_/*K*, where *j *= 0, 1.

We recursively place the sequence **x**_*i *_in all the nodes of T with different weights, starting from the root, based on its estimated cluster membership computed above. Thus, the sequence weight at the root is 1 (all the sequences in the training set lie at the root of the tree), and the weight at any other node in the tree is the product of the sequence weights on the path from the root to that node.

Let $V_{1}^{l},V_{2}^{l},\cdots ,V_{M}^{l}$ be the leaf nodes and $V_{1}^{g},V_{2}^{g},\cdots ,V_{N}^{g}$ be the internal or gating nodes in the hierarchical clustering T. During learning, we train either a collection of *M *Naïve Bayes classifiers or a collection of *M *Logistic Regression classifiers, one classifier at each leaf node $V_{k}^{l}$, *k *= 1,⋯, *M*. Naïve Bayes and Logistic Regression are briefly described in the next section.

To solve the *biomolecular sequence labeling problem*, one approach is to predict each element *x*_*i, j *_in the sequence **x**_*i *_independently, i.e., to assume that the observation-label pairs (*x*_*i, j*_, *y*_*i, j*_)_*j *= 1, *m *_are independent of each other (the *label independence assumption*). However, *x*_*i, j *_may not contain all the information necessary to predict *y*_*i, j*_. Hence, it is fairly common to encode each element *x*_*i, j *_in the sequence **x**_*i *_based on a local, fixed-length window corresponding to the target element and its sequence context (an equal number of its sequence neighbors on each side) ${x^{\prime }}_{i,j}$ = *x*_*i, j*-*t*_,⋯, *x*_*i, j*_,⋯, *x*_*i, j*+*t*_. The classifier is trained to label the target element *x*_*i, j *_[6].

During classification, given a test sequence **x**_*test*_, we extract the local windows corresponding to its elements. Each classifier at the leaf nodes $V_{k}^{l}$ returns the class membership for each window in the test sequence,

$$\begin{array}{cc} p_{V_{k}^{l}}(y_{test,j}=y|{x^{\prime }}_{test,j},x_{test}), & \text{for all }y\in Y \end{array}$$

The gating nodes $V_{k}^{g}$, *k *= 1,⋯, *N *in the hierarchical clustering T combine the predictions of the classifiers to the root node that makes the final prediction. Thus, each gating node combines the predictions from its child nodes (which can be leaf nodes or descendent gating nodes) using the formula:

$$p_{V_{k}^{g}}(y|{x^{\prime }}_{test,j},x_{test})=\sum_{V_{i}\in child(V_{k}^{g})}P_{V_{i}}(y|{x^{\prime }}_{test,j},x_{test})p_{V_{i}}(x_{test}\in V_{i}|x_{test}\in V_{k}^{g})$$

Finally, the window ${x^{\prime }}_{test,j}$ is assigned to the class *y *that maximizes the posterior probability from the root gating node, *V*_*root*_:

$$y=\arg\underset{y\in Y}{\max}p_{V_{root}}(y|{x^{\prime }}_{test,j},x_{test})$$

### Machine learning classifiers

#### Naïve Bayes

Naïve Bayes (NB) [18] is a supervised learning algorithm that belongs to the class of generative models, in which the probabilities *p*(**x**|*y*) and *p*(*y*) of the input **x **and the class label *y *are estimated from the training data using maximum likelyhood estimates. Typically, the input **x **is high-dimensional, represented as a set of features (attributes), **x **= (*x*_1_, *x*_2_, ⋯, *x*_*d*_), making it impossible to estimate *p*(**x**|*y*) for large values of *d*.

However, the Naïve Bayes classifier makes the assumption that the features are conditionally independent given the class:

$$p(x_{1},x_{2},...,x_{d}|y)=\prod_{i=1}^{d}p(x_{i}|y)$$

Therefore, training a Naïve Bayes classifier reduces to estimating probabilities *p*(*x*_*i*_|*y*), *i *= 1,⋯, *d*, and *p*(*y*), from the training data, for all class labels *y*.

During classification, Bayes Rule is applied to compute *p*(*y*|**x**_*test*_):

$$p(y|x_{test})=\frac{p(x_{test}|y)p(y)}{p(x_{test})}$$

The class label with the highest posterior probability is assigned to the new input **x**_*test*_.

#### Logistic Regression

Logistic Regression (LR) [19] is a supervised learning algorithm that belongs to the class of discriminative models. Here, we consider the case of binary classification, where the set of class labels *Y *= {0, 1}. Logistic Regression directly calculates the posterior probability *p*(*y*|**x**) and makes the predictions by threshoding *p*(*y*|**x**). It does not make any assumptions regarding the conditional independence of the features and models the conditional probability of the class label *y *given the input **x **as follows:

$$p(y=1|x;\beta ,\theta )=\frac{1}{1+e^{\left(-\beta^{T}x-\theta \right)}}$$

where [*β, θ*] are the parameters of the model that can be estimated either by maximizing the conditional likelihood on the training data or by minimizing the loss function.

During classification, Logistic Regression predicts a new input **x**_*test *_as 1 if and only if

*β*^*T *^**x**_*test *_+ *θ *> 0

### Ensemble of classifiers

An *ensemble of classifiers *[7,8] is a collection of classifiers, each trained on a *balanced *subsample of the training data (approximately equal number of positive and negative instances obtained by sampling with replacement from the entire training data). The prediction of the ensemble of classifiers is computed from the predictions of the individual classifiers. That is, during classification, for a new unlabeled input **x**_*test*_, each individual classifier in the collection returns a probability *P*_*j*_(*y*_*i*_|**x**_*test*_), that **x**_*test *_belongs to a particular class *y*_*i*_, where *j *= 1,⋯, *m*, and *m *is the number of classifiers in the collection. The ensemble estimated probability, *P*_*Ens*_(*y*_*i*_|**x**_*test*_) is obtained by:

$$P_{Ens}(y_{i}|x_{test})=\frac{1}{m}\sum_{j}^{m}P_{j}(y_{i}|x_{test})$$

In our experiments, we used *m *= 300. Each individual classifier in the collection was trained on approximately $\frac{l}{10}$ instances, where *l *represents the total number of training instances available to the ensemble.

The implementation of all the models considered in this study is built on Weka, an open source machine learning software available at [20].

### Performance evaluation

To assess the performance of classifiers in this study, we report the following measures: Precision, Recall, Correlation Coefficient (CC), and F-Measure (FM). If we denote true positives, false negatives, false positives, and true negatives by *TP, FN, FP*, and *TN *respectively, then these measures can be defined as follows:

$$\text{Precision}=\frac{TP}{TP+FP}$$

$$\text{Recall}=\frac{TP}{TP+FN}$$

$$\text{CC}=\frac{TP\cdot TN-FP\cdot FN}{\sqrt{\left(TP+FN)(TP+FP)(TN+FP)(TN+FN\right)}}$$

$$\text{FM}=\frac{2\times \text{Precision}\times \text{Recall}}{\text{Precision}+\text{Recall}}$$

To obtain the estimates for *TP, FN, FP *and *TN*, we performed 10-fold sequence-based cross-validation [21] wherein the set of sequences is partitioned into 10 disjoint subsets (folds). At each run of a cross-validation experiment, 9 subsets are used for training and the remaining one is used for testing the classifier. The values for *TP, FN, FP *and *TN *are obtained using the default threshold *θ *= 0.5, i.e., an instance is classified as positive if the probability of being in the positive class returned by the classifier is greater than or equal to 0.5, and as negative otherwise.

With any classifier, it is possible to tradeoff the Precision against Recall. Hence, it is more informative to compare the Precision-Recall curves which show the tradeoff over their entire range of possible values than to compare the performance of the classifiers for a particular choice of the tradeoff.

The Precision-Recall curve is a good indicator of the performance of classifiers when the data sets are highly unbalanced, as is the case with our both RNA- and DNA-protein data sets [22]. It has also been shown that if a curve dominates in PR space, it also dominates in ROC space [22].

To evaluate how good a classifier is at discriminating between the positive and negative examples, we also report the Area Under the Receiver Operating Characteristic Curve (AUC) on the test set, which represents the probability of correct classification [23].

## Competing interests

The authors declare that they have no competing interests.

## Authors' contributions

CC and JS carried out the computations. CC prepared an initial draft of the manuscript. All authors participated in experimental design, discussions, and manuscript preparation. All authors read and approved the final manuscript.

## References

[B1] Terribilini M, Lee JH, Yan C, Jernigan RL, Honavar V, Dobbs D (2006). Predicting RNA-binding Sites from Amino Acid Sequence. RNA Journal.

[B2] Yan C, Dobbs D, Honavar V (2004). A Two-Stage Classifier for Identification of Protein-Protein Interface Residues. Bioinformatics.

[B3] Qian N, Sejnowski T (1988). Predicting the secondary structure of globular proteins using neural networks models. J Mol Biol.

[B4] Caragea C, Sinapov J, Silvescu A, Dobbs D, Honavar V (2007). Glycosylation site prediction using ensembles of Support Vector Machine classifiers. BMC Bioinformatics.

[B5] Kim JH, Lee J, Oh B, Kimm K, Koh I (2004). Prediction of phosphorylation sites using SVMs. Bioinformatics.

[B6] Diettrich TG (2002). Machine Learning for Sequential Data: A Review. Proceedings Joint IAPR International Workshop on Structural, Syntactic, and Statistical Pattern Recognition.

[B7] Dietterich TG (2000). Ensemble Methods in Machine Learning. Lecture Notes in Computer Science.

[B8] Russell S, Norvig P (2003). Artificial Intelligence: A Modern Approach.

[B9] Jordan MI, Jacobs RA (1994). Hierarchical mixtures of experts and the EM algorithm. Neural Computation.

[B10] Dempster AP, Laird NM, Rubin DB (1977). Maximum likelihood from incomplete data via the EM algorithm. Journal of the Royal Statistical Society.

[B11] Berman H, Westbrook J, Feng Z, Gilliland G, Bhat T, Weissig H, Shindyalov I, Bourne P (2000). The Protein Data Bank. Nucleic Acid Res.

[B12] Allers J, Shamoo Y (2001). Structure-based analysis of protein-RNA interactions using the program ENTANGLE. J mol Biol.

[B13] Using BLASTClust to Make Non-redundant Sequence Sets.

[B14] Duda R, Hart E, Stork D (2001). Pattern Classification.

[B15] Shi J, Malik J (2000). Normalized cuts and image segmentation. Pattern Analysis and Machine Intelligence.

[B16] Dhillon IS (2001). Co-clustering documents and words using bipartite spectral graph partitioning. Proceedings of SIGKDD International Conference on Knowledge Discovery and Data Mining.

[B17] Paccanaro A, Casbon JA, Saqi MAS (2006). Spectral clustering of protein sequences. Nucleic Acids Research.

[B18] Mitchell TM (1997). Machine Learning.

[B19] Ng AY, Jordan MI (2002). On discriminative vs. generative classifiers: A comparison of logistic regression and naive Bayes. Advances in Neural Information Processing Systems (NIPS), NIPS.

[B20] Weka 3: Data Mining Software in Java. http://www.cs.waikato.ac.nz/ml/weka/.

[B21] Caragea C, Sinapov J, Dobbs D, Honavar V (2007). Assessing the Performance of Macromolecular Sequence Classifiers. IEEE 7th International Symposium on Bioinformatics and Bioengineering.

[B22] Davis J, Goadrich M (2006). The Relationship Between Precision-Recall and ROC Curves. Proceedings of the 23rd International Conference on Machine Learning.

[B23] Baldi P, Brunak S, Chauvin Y, Andersen C, Nielsen H (2000). Assessing the accuracy of prediction algorithms for classification: an overview. Bioinformatics.

